# Applications of Microscope-Integrated Indocyanine Green Videoangiography in Cerebral Revascularization Procedures

**DOI:** 10.3389/fsurg.2019.00059

**Published:** 2019-11-28

**Authors:** Claudio Cavallo, Sirin Gandhi, Xiaochun Zhao, Evgenii Belykh, Daniel Valli, Peter Nakaji, Mark C. Preul, Michael T. Lawton

**Affiliations:** Department of Neurosurgery, St. Joseph's Hospital and Medical Center, Barrow Neurological Institute, Phoenix, AZ, United States

**Keywords:** indocyanine green videoangiography, cerebral revascularization, extracranial-intracranial bypass, intracranial-intracranial bypass, graft patency

## Abstract

Indocyanine green videoangiography (ICG-VA) is a near-infrared range fluorescent marker used for intraoperative real-time assessment of flow in cerebrovascular surgery. Given its high spatial and temporal resolution, ICG-VA has been widely established as a useful technique to perform a qualitative analysis of the graft patency during revascularization procedures. In addition, this fluorescent modality can also provide valuable qualitative and quantitative information regarding the cerebral blood flow within the bypass graft and in the territories supplied. Digital subtraction angiography (DSA) is considered to be the gold standard diagnostic modality for postoperative bypass graft patency assessment. However, this technique is time and labor intensive and an expensive interventional procedure. In contrast, ICG-VA can be performed intraoperatively with no significant addition to the total operative time and, when used correctly, can accurately show acute occlusion. Such time-sensitive ischemic injury detection is critical for flow reestablishment through direct surgical management. In addition, ICG has an excellent safety profile, with few adverse events reported in the literature. This review outlines the chemical behavior, technical aspects, and clinical implications of this tool as an intraoperative adjunct in revascularization procedures.

## Introduction

Revascularization procedures are considered in the management of various cerebrovascular disorders, including complex intracranial aneurysms, moyamoya disease (MMD), symptomatic cerebral ischemia recalcitrant to standard medical therapy, and cranial base tumors that require a proximal major vessel sacrifice. MMD is a pathological condition that is characterized by a bilateral progressive narrowing at the terminal portion of the intracranial internal carotid arteries. This stenosis may lead to occlusion of the vessel lumen and a consequent abnormal, compensatory collateral neovascularization at the base of the brain. Superficial temporal artery (STA)-middle cerebral artery (MCA) bypass is the main direct revascularization procedure performed, and it is effective in reducing the risk of ischemic and hemorrhagic strokes as well as in improving cerebral ischemia (1–3).

Microscope-integrated indocyanine green videoangiography (ICG-VA) is a relatively recent technique used as an intraoperative adjunct for real-time assessment of anastomotic patency. Indocyanine green was approved by the US Food and Drug Administration (FDA) in 1956 for the evaluation of cardio-circulatory and liver functions. In addition, the FDA approved ICG for use in ophthalmic angiography in 1975 (4). Since then, ICG has been extensively used for retinal angiography (5, 6). Raabe et al. revived this technique to document intravascular flow in cerebral vessels to establish vascular patency (4). ICG-VA was also used for the intraoperative assessment of dural arteriovenous fistulas, for confirming complete aneurysm obliteration, and avoiding perforator or branch vessel compromise on microsurgical clipping (7). Over the years, ICG-VA has become an effective low-cost, non-invasive technique for confirming real-time intravascular flow in bypasses, with a good safety profile. The objective of our study was to present a focused review of the application of ICG-VA in cerebral bypass procedures and to describe the current diagnostic and therapeutic modalities of this technique.

## Chemical Properties, Pharmacokinetics, and Adverse- Effect Profile of ICG

ICG (C_43_H_47_N_2_NaO_6_S_2_) is a tricarbocyanine dye with near-infrared fluorescent properties.

The absorption and emission peaks of ICG are 805 and 835 nm, respectively, which fall in the near-infrared (NIR) light range. The NIR peaks allow greater transmission of the fluorescence signal through the native tissue compared with the fluorophores, with peaks in the visible light range (6, 8–10). In theory, ICG fluorescence has a penetration depth of 10–20 mm in human tissues (11). This two-dimensional fluorescence imaging provides high spatial resolution and image quality compared with other intraoperative techniques.

On intravenous administration, ICG binds to alpha-1 lipoproteins, belonging to the globulin family, and remains intravascular as long as the vascular wall integrity is preserved. This fluorescent compound does not enter the enterohepatic circulation and is not metabolized in the body. It has a plasma half-life of 3–4 min and is excreted exclusively by the liver. Despite its relatively good safety profile, the ICG molecule or the 5% sodium iodine contained in the drug can potentially cause adverse reactions (4). Mild reactions, such as nausea, vomiting, pruritus, and sneezing, have been reported in ~0.15% of cases. Severe adverse reactions, including bronchospasm, laryngospasm, anaphylaxis, shock, myocardial infarction, cardiovascular arrest, and tonic clonic seizures have occurred in ~0.05% of cases (4). Overall, ICG is associated with a low rate of adverse reactions, comparable to other contrast media. Iodine allergy or prior adverse reactions to contrast media are contraindications for the administration of ICG.

## Technical Aspects of ICG-VA

Intraoperative technology for visualization of fluorescence is an integrated component of the currently available surgical microscopes. An excitation light in the NIR range is transmitted through the microscope optics to generate ICG fluorescence. This emitted electromagnetic radiation is invisible to the human eye and passes through the NIR optical filter (INFRARED 800. Zeiss AG, Oberkochen, Germany) that blocks both the excitation and the ambient light. An infrared-sensitive camera on the surgical microscope detects the ICG fluorescence and translates the signal into a real-time white image on a black background. Recently, an innovative dual-image videoangiography system was developed to overlay visible light and NIR fluorescence images and allow the simultaneous intraoperative visualization of both ICG fluorescence and surrounding anatomical structures (12).

Before activating the INFRARED 800 mode to perform ICG-VA, the focus distance and the magnification should be set at <300 mm and less than 5×, respectively. Afterward, the surgeon can adjust these two parameters during the INFRARED 800 recording to optimize the image. However, modern surgical microscopes usually assist the user with an automatic function that fine-tunes the settings to render the highest quality images. The KINEVO 900 (Carl Zeiss) provides an AutoZoom function that automatically adjusts the total magnification and diaphragm opening to the preconfigured values at the start of ICG-VA and an AutoGain function that automatically adjusts the camera gain to fit the NIR signal intensity within the dynamic range of the camera as the ICG-VA progresses. Chromatic aberration due to higher magnification and surgical field depth account for the inability of electromagnetic radiation of different wavelengths to focus on the same positions in the focal plane. Through the use of special lenses, apochromatic optics adjust light arrays of different colors to converge to the same focal point. This method achieves a variable degree of compensation that is more significant in the visible light spectrum (400–700 nm). Chromatic aberration of NIR light, like the ICG emission light (820–900 nm) can be compensated the most at working distances of <300 mm and at low magnification (<5×). The use of zoom and focal values other than those recommended can increase the focal point discrepancy between the NIR and visible light images and affect the quality of ICG-VA, resulting in a blurry NIR image.

The usual dose of ICG used is 0.2–0.5 mg/kg. At the time of administration, 25 mg of the dye is dissolved in 5 mL water and flushed with 10 mL of normal saline as a single intravenous bolus. Once the INFRARED 800 mode is activated, the ICG solution is injected through a peripheral line. ICG can cause lower values of peripheral oxygen saturation to be falsely displayed. The surgeon should manipulate and mobilize anatomical structures through the operating oculars of the surgical microscope to ensure that the bypass graft is visible. Although NIR fluorescence has significantly greater penetration depth than visible light, the blood flow can be appreciated only in exposed blood vessels that are not obscured by anatomical structures, brain parenchyma, surgical instruments, or patties. The ICG fluorescence signal cannot be visualized directly through the operating oculars but is seen on the monitor of the surgical microscope following image processing (13, 14). The operating neurosurgeon can manipulate the vessels while the assistant reports the ICG-VA findings displayed on the screen with the ICG module activated.

An ICG-VA image takes ~10–30 s to appear on the screen. Once the recording is ended by the user, the automatic playback mode of the operating microscope allows the surgeon to assess the quality of the imaging in detail. ICG-VA can be repeated over time without any significant interference from the residual fluorescence of the previous injection. However, ICG should be reinjected after 20 min to guarantee adequate clearance of the tracer and optimize contrast enhancement (13, 14). Repeated administration of the dye should not exceed a total cumulative dose of 5 mg/kg.

ICG-VA can also analyze the intraluminal flow rate and flow velocity in selected regions of interest. In addition, a graph is generated to display the results of this quantitative analysis in a map delay time. The time characteristics of the graph allow the evaluation of the time to peak, defined as the interval from uprise of a wave to highest signal intensity, and washout time, represented by the interval from uprise of a wave to the time of rapid fall of signal intensity.

## Application of ICG-VA

### Assessment of Graft Patency

During bypass surgery, the assessment of intravascular graft patency is essential to reduce the rate of graft failure. Early intraoperative identification of an occluded graft artery is critical for prompt surgical correction and for early restoration of blood flow to ensure a successful bypass. Several characteristics of the donor and graft vessels have been found to affect bypass function, including the graft diameters and the extent of focal atherosclerotic change (15). Other critical factors that play a role in intraoperative bypass patency include the cerebral blood flow demand of the downstream vascular territory and the direction of blood flow—antegrade vs. retrograde (15, 16). Technical aspects related to the surgical procedure or hypercoagulable states may also be responsible for the occurrence of a graft occlusion (17). Early intraoperative detection of an immediate bypass occlusion using ICG allows us to potentially identify and restore the patency of the bypass to prevent iatrogenic brain ischemia. Early detection may also impact the functional outcome for the patient and decrease the morbidity and mortality associated with revascularization procedures.

Direct intraoperative visual inspection of the anastomotic site cannot always accurately determine the patency of the graft vessel. The postoperative graft patency rate reported in the existing literature for an extracranial-intracranial (EC-IC) bypass is 87–96% (18–22). However, the aforementioned rate varies depending on the intraoperative diagnostic tool used to detect this complication. There are numerous intraoperative monitoring techniques for the evaluation of the graft patency. Among all of them, digital subtraction angiography (DSA) is considered to be the gold standard (23). Despite its diagnostic accuracy in identifying an intraoperative graft occlusion, this diagnostic imaging technique has several limitations, including its invasive nature and the associated risks; high procedural costs; the need for dedicated, trained personnel; and exposure of the patient to prolonged ionizing radiation (18). Moreover, DSA is time-consuming and detects the vessel occlusion in a delayed fashion, in contrast to ICG, which allows for an intraoperative diagnosis and immediate intervention. Other intraoperative monitoring techniques include Doppler ultrasonography (24) and thermal artery imaging (25). Intraoperative microvascular Doppler ultrasonography can be done directly on the bypass graft to assess its patency and quantitatively evaluate the blood flow metrics (26–28). The blood flow values can then be used to calculate the intraoperative cut flow index (CFI), which is the ratio of flow through the graft to the flow through the donor vessel prior to bypass (cut flow). CFI seems to be a sensitive parameter for postoperative bypass patency (29). Indeed, a previous study evaluating 51 bypass procedures for flow augmentation demonstrated that a CFI <0.5 and CFI > 0.5 were associated with patency rates of 50 and 92%, respectively (28). Alternatively, an ultrasound system with a multifrequency linear probe can be used to perform a standard B-mode acquisition and a color Doppler evaluation with or without ultrasound contrast agents. This technique allows the intraoperative assessment of the arterial course and flow through the intracranial vessels present in the depth of the surgical field, which are deeper than the cortical surface in contact with the probe (30).

However, these techniques have major drawbacks in terms of spatial resolution and image quality, such that blood flow in small perforating arteries cannot be evaluated.

MR and CT angiography are effective non-invasive radiological modalities for evaluation of the patency of the bypass in the early postoperative period (18). Conventional DSA can be employed in the postoperative period to obtain valuable information regarding the graft patency and the dynamics of the bypass functionality (23). Other diagnostic adjuncts like functional cerebral blood flow (CBF) studies, SPECT, and Xe-enhanced CT are indicated to assess the postoperative cerebral perfusion.

As mentioned earlier, ICG-VA is a cost-effective and easy-to-use technique that provides real-time visualization of the fluorescent tracer inflow within cerebral vessels. ICG-VA is ideal for the intraoperative assessment of graft patency, and it seems to be superior to standard angiographic techniques (18). It consists of three different phases: arterial, capillary, and a combined arteriovenous phase generated by the recirculation of the dye. The early arterial phase is the most critical to detect delay or cessation of flow in the bypass graft, arterial branches, or perforators (4). Surgical microscope settings for fluorescence detection might differ substantially from the standard ones adopted for intraoperative visualization under conventional white light. Indeed, flow in perforating vessels is better visualized with a lower magnification and a shorter focus distance (4).

The remarkable spatial resolution of ICG-VA allows assessment of the morphology of graft patency and the hemodynamic of the bypass functioning (18). This diagnostic information is based on the pattern of filling of the graft with the fluorescent tracer compared with the contrast enhancement of the surrounding cerebral blood vessels. Moreover, this property allows the localization of the graft occlusion in real time in case of early bypass failure (13). Revision of the bypass graft might resolve this early intraoperative complication and improve surgical results related to revascularization procedures. Several clinical factors may influence the image quality with ICG-VA, such as the cardiovascular dynamics and cerebrospinal fluid (CSF) accumulation. The technical factors that qualitatively alter ICG-VA images include the speed of ICG injection and microscope settings, such as light intensity, working distance, magnification, and the diameter of the luminous field diaphragm opening. A previous study reported that ICG-VA image quality was notably affected by high magnification and depth of field based on chromatic aberration, as previously elaborated (13, 14).

In a previous series, Woitzik et al. reported bypass function in 31 of 35 (89%) STA-MCA bypasses, 2 of 2 (100%) STA-PCA bypasses, and 6 of 8 (75%) high-flow bypasses (18). Four STA-MCA bypasses had intraoperative occlusion, whereas 2 saphenous vein high-flow bypasses demonstrated moderate graft filling. In all these cases, ICG-VA localized the site of bypass failure at the anastomotic site, and timely surgical revision restored the flow through the bypass. Patency of the graft was subsequently documented by postoperative DSA or CT angiography.

### Intraoperative Flow Analysis

Januszewski et al. described three different types of flow through a graft: Type I, robust anterograde flow; Type II, delayed anterograde flow; and Type III, anterograde delayed flow but with no continuity to the bypass site or no convincing flow. This study analyzed 36 EC-IC and intracranial-intracranial (IC-IC) bypasses performed in 33 ischemic patients with hemodynamic compromise despite maximal medical therapy (7). In their cohort, 8% of patients with Type II flow on ICG-VA had CTA findings indicating a graft occlusion within 72 h. Interestingly, none of the patients with Type II or Type III flow pattern had any clinical or radiological evidence of stroke in the postoperative period. This finding may be justified by the presence of competitive collateral flow leading to a lack of demand through the graft. Type III flow can also be caused by either technical surgical complications at the anastomosis or poor arterial graft quality and is associated with a higher probability of eventual graft failure (7). In contrast, failure in a Type II flow pattern is less likely to be related to the presence of competitive regional flow from collateral vessels. Therefore, the authors recommend reexploration of anastomoses in a Type II flow category to detect any early signs of bypass failure with the goal of immediate revision to salvage the bypass. If the quantitative flow characterization on ICG-VA images can be standardized for use across various medical centers, it would overcome a possible observer bias (7). Despite the results of this pilot study, a correlation between the flow characterization and the eventual clinical outcome is yet to be proven.

A quantitative analysis of flow was performed by Awano et al. using a specialized image analysis software (17). The ICG signal was quantified and the perfusion area of ICG was calculated at the point of maximal fluorescence intensity in specific regions of interest on the cortical surface (17). Awano et al. developed an innovative system based on visual light spectroscopy to continuously record hemodynamic changes of cortical blood flow during STA-MCA bypass surgery (17). Two groups of patients were included in this study, patients with and patients without MMD (with other indications for revascularization). The authors demonstrated that the ICG perfusion area was larger with a significant increase in cortical oxygen saturation in the MMD cohort. This could be attributable to the increase of blood flow demand and the larger pressure gradient between the anastomosed STA and the recipient vessels.

### ICG-VA Role in Predicting Postoperative Neurological Complications

In recent years, several studies investigated the role of semi-quantitative and quantitative analysis of intraoperative ICG-VA findings in predicting the occurrence of postoperative neurological events after revascularization surgery in MMD. A variety of transient neurological events (TNEs) have been reported in the immediate postoperative period after STA-MCA bypass, which is most commonly employed in MMD treatment. TNEs occur in 14–77% of patients after MMD bypass surgery (3) and include extremity numbness, weakness, and episodic severe headaches (31–34), which could potentially have permanent neurological sequelae (31–34). The underlying causative mechanisms of TNEs are yet to be elucidated. However, the postoperative hemodynamic changes after revascularization procedures suggest that these complications might be related to local cerebral hyperperfusion (HP) (31, 35) or regional inflammation or due to a watershed shift (33, 36). In addition, extravasation of contrast materials in radiological imaging was found to be associated with cerebral HP after revascularization, suggesting a possible role for the breakdown of the blood-brain barrier (37, 38). Horie et al. described the diagnostic criteria for postoperative symptomatic HP as (1) the presence of focal neurologic deficits and/or severe headache, (2) confirmed graft patency by magnetic resonance angiography, (3) evident postoperative increase in CBF, and (4) absence of any ischemic changes and other pathologies (31, 32, 39–41). Postoperative HP occurs more frequently in adults than in pediatric patients in MMD (29, 31, 35). Postoperative radiological HP has been reported to occur in ~60% of the adult MMD population overall, and ~30% of the adult MMD population experiences symptomatic HP (35). Some diagnostic adjunct intraoperative laser Doppler or thermal diffusion probe (42) can detect an increase in intraoperative CBF in the anastomotic site, indicative of postoperative HP (43, 44). In addition, the use of infrared imaging, electromagnetic flowmetric, and Adobe Photoshop (Adobe Inc., San Jose, California) software have also been reported for the evaluation of hemodynamics (43–45). In patients undergoing a STA-MCA bypass for intracranial atherosclerotic disease or symptomatic carotid stenosis, a reduction in CBF and cerebrovascular reserve can be predictive of postoperative HP. However, this phenomenon does not apply to MMD patients (35, 41). In their prospective study, Horie et al. performed ICG-VA after STA-MCA anastomosis and compared cortical perfusion between the adult and pediatric populations with MMD as well as in adult patients with atherosclerotic disease (46). On evaluation of ICG intensity-time curves of the donor (STA), recipient (MCA), and the surrounding vessels, the postoperative HP was assumed to depend on the donor vessel caliber and on the poor integrity of the blood-brain barrier in the recipient. In this context, ICG-VA can be potentially utilized to predict postoperative HP in MMD patients. Similarly, a study by Uchino et al. suggested that semiquantitative analysis of ICG-VA is useful in predicting potential occurrence of HP after STA-MCA anastomosis in occlusive carotid artery disease (47). ICG-VA is effective in assessing intraoperative hemodynamic changes. Moreover, the blood flow index, calculated on the basis of the ICG intensity-time curve, can document the augmentation of cortical perfusion. A blood flow index increase greater than 3-fold from the baseline preoperative value may predict the occurrence of HP, whereas repeated SPECT is necessary to diagnose delayed postoperative HP.

Similarly, Uda et al. analyzed ICG-VA using specialized software (FlowInsight, Infocom Corporation, Shibuya, Tokyo, Japan) that allows the recording of several parameters in different regions of interest around the anastomotic site, including CBF, mean transit time, mean gradation time (Grad), and time to peak (TTP). Percentage change of these parameters after bypass was calculated using the formula (ΔX = (Xafter - Xbefore)/Xbefore). They demonstrated that patients with ΔTTP ≤ −12, ΔCBF ≥ 8, or ΔGrad ≥ 30 have a higher risk of developing postoperative TNEs (3). Hemodynamic changes after bypass surgery, analyzed through ICG and FlowInsight, were predictive of postoperative TNE and correlated with their duration. Optimization of the clinical management of these patients may further reduce TNE-related complications after revascularization procedures. Despite several reports demonstrating the role of hemodynamic changes detected by ICG-VA image analysis in potentially predicting postoperative HP and TNEs, there is no current evidence showing a correlation with improved treatment outcomes. Moreover, the role of ICG-VA in predicting delayed postoperative HP several days after surgery remains uncertain (47). Larger studies are warranted to confirm these results and further elucidate the contribution of this fluorescent intraoperative technique.

### Intraoperative Vessel Identification

EC-IC bypass surgery, in particular STA-MCA anastomosis, is the most common revascularization procedure for the treatment of cerebral ischemia. Designed and successfully performed for the first time by Yaşargil in 1967 (48–50), this surgery is aimed at providing alternative blood supply in patients with an underlying hemodynamic cerebrovascular insufficiency (51). Visualization of STA and its branches is critical, and an adequate donor artery can be selected preoperatively using different imaging modalities, including DSA, CTA, and magnetic resonance angiography (49). In addition, the course of STA from the underside of the frontotemporal skin flap can be mapped intraoperatively by light palpation, continuous Doppler ultrasound, or through the assistance of frameless electromagnetic neuronavigation to guarantee a safe dissection (49, 52). The parietal branch of STA is the more commonly used donor graft. However, in certain cases with a planned double-barrel STA-MCA bypass or with an atrophic or damaged parietal STA, the frontal branch of the artery is used. ICG-VA can aid in the intraoperative visualization of STA and assist in the identification of the donor artery, especially when the anterior frontal branch is selected. In addition, ICG-VA provided valuable intraoperative information for the mapping and preparation of the donor vessel (50).

Peña-Tapia et al. first described a new application of ICG-VA to identify a cortical target point and find an adequate recipient vessel (51). They used a rectangular template made of transparent plastic material that can be applied to both sides of the head and with a handle centered on the outer auditory canal. This template-based approach allows the preoperative identification of the end of the sylvian fissure, accurately relying on external landmarks (51). Two different highlighted lines are engraved on the surface of the plastic, and their overall orientation is aimed at identifying several M4 (segment of the MCA) branches emerging from the sylvian fissure, which can be selected as recipient vessels and marked on the patient's scalp.

Previous studies have shown that the application of ICG-VA is not limited to the identification or safe dissection of the STA in bypass procedures. In fact, this intraoperative technique has also been used to selectively target the most suitable cortical M4 recipient artery for flow-preserving bypass to trap complex MCA aneurysms (53). Alternatively, M2-M3 segments of the MCA could also be selected if the anatomy is favorable and the microsurgical dissection of the sylvian fissure is safe (53). However, M2-M3 segments may feed cortical M4 vessels and the STA-MCA anastomosis may redirect the blood supply to uninvolved vascular territories despite the use of anatomical landmarks, neuroimaging, and neuronavigation (52, 54). For this reason, a terminal branch of the MCA (M4) is preferred as a recipient vessel. Moreover, temporary ischemia is considered less invasive and is better tolerated in M4 superficial cortical arteries than in M2-M3 segments (54, 55). During intraoperative ICG-VA, the cortical surface in the surrounding area of the sylvian fissure is analyzed to detect the direction of the flow and possible delayed or reversed filling time of M4 branches during the arterial phase and other vascular structures during the capillary and venous phase (52, 54). Esposito et al. described a primary and a secondary identification in their surgical protocol for intraoperative ICG-VA (53). The primary identification consists of performing a baseline ICG-VA without any temporary occlusion of arterial vessels. On the contrary, the secondary identification consists of intraoperative ICG-VA with a “provocative” temporary occlusion. Delayed fluorescent filling of superficial M4 branches during either primary or secondary identification demarcates the cortical territory supplied, and any artery confined within this area can be selected as the recipient vessel (53). ICG-VA can be repeated after anastomosis to rule out any residual filling in the complex MCA aneurysm.

**Figure 1:**
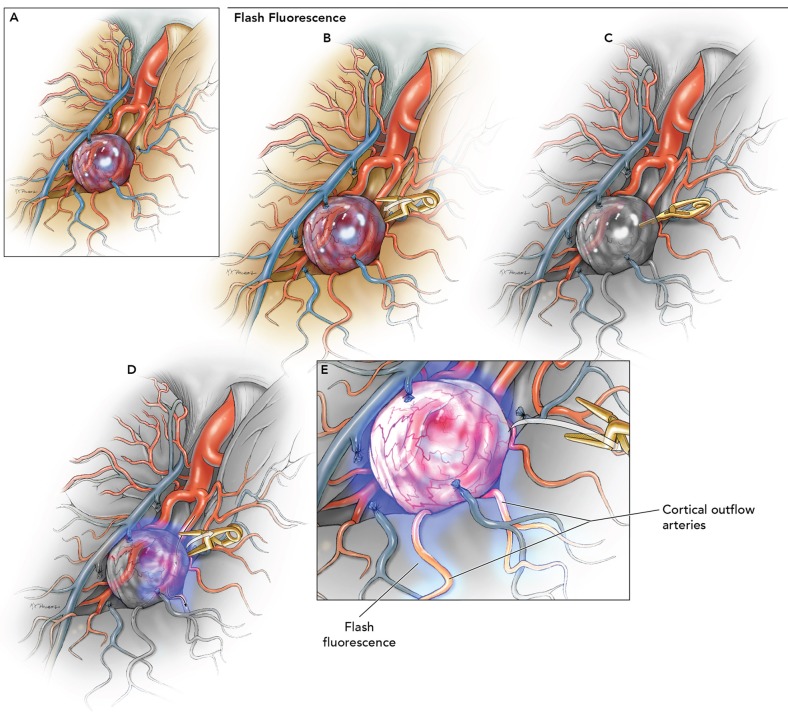
Use of flash fluorescence technique to identify the efferent arteries of the aneurysm using ICG-VA. **(A)** Identification of candidate bypass recipient arteries among the surface M4 branches is difficult but could be improved using the following steps. **(B)** Temporary clip occlusion of the aneurysm inflow (afferent arteries) proximal to the aneurysm. **(C)** ICG-VA demonstrating initial fluorescence in uninvolved arterial branches. **(D)** Removal of temporary clip for aneurysm reperfusion. **(E)** Fluorescence seen in the efferent arteries to identify the most suitable recipient on the cortical surface for the bypass. Used with permission from Lawton (56).

**Figure 2:**
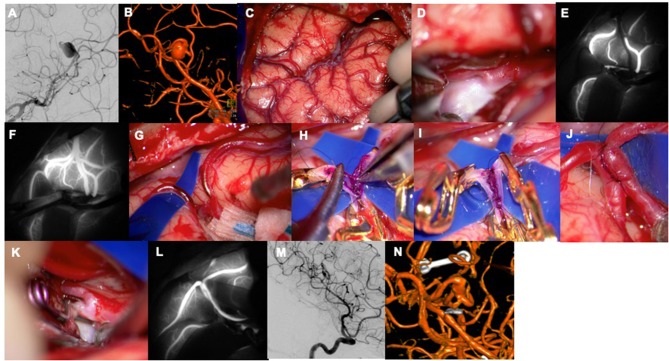
A 47-year-old woman presented with a sudden headache suspicious for sentinel hemorrhage. **(A)** An aneurysm was found at the distal M2 segment with dolichoectatic morphology and two efferent branches (left ICA, AP view). **(B)** Left ICA, 3D reconstruction. **(C)** Sylvian fissure split to access the insular recess. **(D)** Aneurysm with parent artery. **(E)** Flash fluorescence technique was used to identify the efferent vessel to be used as a bypass recipient with proximal temporary clip occlusion. **(F)** Temporary clip removal led to flash ICG filling of this vascular territory and showed the angular artery as the outflow vessel. **(G)** *In situ* bypass of angular artery with posterior temporal artery was performed. **(H)** The intraluminal suture line was sewn with a single anchoring stitch to better visualize the walls. **(I,J)** The extraluminal suture line was sewn loosely, tightened, and tied. **(K)** Proximal aneurysm occlusion. **(L)** M3 MCA-M3 MCA *in situ* bypass perfused the donor angular artery as seen with ICG-VA. **(M)** Left ICA, lateral view. **(N)** Left ICA, 3D reconstruction. Used with permission from Lawton (56).

The senior author has previously introduced the “flash fluorescence” technique (Figures 1, 2) to select an adequate recipient artery for bypass. The application of this technique is summarized in the three following surgical steps: (1) proximal temporary occlusion of the afferent artery of the aneurysm, (2) ICG-VA to define the uninvolved cortical arteries, and (3) temporary clip removal for reperfusion of the afferent artery and concomitant flash fluorescence ICG run in efferent arteries on the cortical surface (57). The recipient artery is initially dark during the ICG-VA and flash fluorescence. This direct technique enables the selection of the distal recipient artery. An alternative, indirect technique has been described that relies on temporary occlusion of the uninvolved arteries adjacent to the aneurysm's afferent artery. In this case, the efferent arteries illuminate first and simultaneously flash fluorescence with temporary clip removal to highlight the arteries that should not be considered as recipients for the bypass. The direct technique is preferred, as it requires a more limited ischemia time and only one temporary clip. Flash fluorescence is especially valuable with distal MCA aneurysms that require bypass because the identification of a distal cortical recipient prevents unnecessary sylvian fissure dissection. Moreover, brain retraction is not needed, and the anastomosis is more easily performed on the cortical surface than in the deep sylvian location. Compared with the conventional pterional craniotomy, the bone flap should be extended posteriorly to accommodate the more distal location of the anastomotic site. Other authors reported the use of transdural ICG-VA in STA-MCA bypass procedures to expose the appropriate cortical recipient distributing from the sylvian fissure and superficial vessels anatomy. The location of the recipient was then marked on the dura mater with pyoktanin blue to tailor the dural incision and prevent possible damage during dural opening and microsurgical dissection (58). ICG-VA has also been used to trace the course of the middle meningeal artery (MMA) for use in indirect bypass, encephalo-duro-myo-arterio-pericranial synangiosis for MMD (59). This MMA anterior branch provides collateral flow through the dura mater to the anterior cerebral artery territory both spontaneously and through indirect bypass (60, 61). The standard fronto-temporal craniotomy can damage the anterior branch of MMA given its course within the lesser wing of the sphenoid bone in 50–75% of patients (62, 63). Based on the ICG-VA findings, the craniotomy has been customized into a heart-shaped bone flap to prevent damage to the anterior MMA.

## ICG-VA vs. Fluorescein Videoangiography

Fluorescence videoangiography has gained significant traction in the recent years in the practice of complex cerebrovascular neurosurgery. Despite the widespread acceptance of ICG-VA in neurosurgical procedures, the recent reemergence of fluorescein sodium as a fluorescent tracer has prompted authors to investigate and compare fluorescein videoangiography (FL-VA) with ICG-VA. An objective analysis was recently conducted by applying quantitative metrics to data derived from video capture images, which were subsequently elaborated by pixel intensity-analyzing software (62). In this fluorescence study, ICG-VA demonstrated a greater potential for allowing the robust visualization of the extracranial arteries, including the STA and thick intracranial vessels. On the contrary, FL-VA provided superior intraoperative information regarding perforating arteries, distal branches, and small vessels at high magnification deep in the surgical field (62). In addition, FL-VA allows the surgeon to perform a real-time flow assessment and manipulation of vessels of interest by direct intraoperative visualization through the operating microscope oculars.

The widespread acceptance of ICG-VA is attributable to its ease of use along with its diagnostic accuracy, low cost, and good safety profile (64). Another important advantage of ICG-VA is the ability to perform repeated injections with a short refractory period for intraoperative blood flow reassessment (64). The limitations of fluorescein in vascular neurosurgery are mainly related to its tendency to leak more readily into the extravascular space, resulting in a more persistent fluorescent signal in the surgical field that prevents repeated injections (64). Despite their differences, these two fluorescent modalities are complementary in nature, and their application should be based on the surgical procedure, the depth of the operative field, and the size of the vessels of interest.

## Future Direction for Application of ICG-VA

In recent years, a few novel techniques in the application of ICG-VA have been described. These techniques include the introduction of a “second window” ICG technique for glioblastoma resection that capitalizes on the inherent property of permeability of the tumor vasculature. A high dose of ICG is delivered ~24 h before surgery, leading to non-specific accumulation of the tracer within the tumor tissue. This technique may allow real-time intraoperative tumor identification to optimize surgical resection (65). Additionally, ICG-VA has been used to identify the venous drainage pattern and collateral circulations during tumor resection for possible venous sacrifice to predict and minimize the potential side effects (66). However, the role of these techniques in the setting of revascularization has yet to be elucidated.

### Other Applications of ICG-VA

The contribution of ICG-VA has also been investigated for other types of revascularization procedures including coronary artery bypass. The calculated sensitivity and specificity of ICG in detecting 50% stenosis were 83.3 and 100%, respectively (67). However, ICG-VA is associated with several limitations that preclude its widespread acceptance and use in this subspecialty, in contrast to neurosurgery. ICG visualization of pedicle conduits is poorer when compared with skeletonized conduits, and each graft requires up to 4 min to be evaluated, resulting in a significant effect on the operative time (68). Moreover, the “semi-quantitative assessment” of graft patency with ICG-VA provides inadequate information on the distal coronary vasculature as well as competitive flow and transit times (69). Despite other studies showed promising results, the use of ICG for detecting coronary bypass patency has yet to be definitively proven (70).

## Conclusion

ICG-VA has been widely established as a useful technique to perform qualitative analyses of graft patency and CBF during revascularization procedures. The widespread incorporation of ICG-VA is attributable to its ease of use, diagnostic accuracy, low cost, and excellent safety profile.

## Author Contributions

CC: literature review, manuscript draft, revision, and final draft approval. SG: manuscript draft, revision, and review of final manuscript. XZ and EB: manuscript draft and review of final draft. DV: literature review and final draft review. PN, MP, and ML: study supervision, review, and revision of the final draft.

### Conflict of Interest

The authors declare that the research was conducted in the absence of any commercial or financial relationships that could be construed as a potential conflict of interest.

## Abbreviations

CBF: cerebral blood flow
CFI: cut flow index
CSF: Cerebrospinal fluid
DSA: Digital subtraction angiography
EC-IC: Extracranial-intracranial
FDA: US Food and Drug Administration
HP: hyperperfusion
ICG-VA: indocyanine green videoangiography
IC-IC: Intracranial-intracranial
MCA: Middle cerebral artery
MMD: Moyamoya disease
NIR: near infrared
PCA: Posterior cerebral artery
STA: Superficial temporal artery
TNE: transient neurological event.
